# Supplementary material for: Splice-Junction-Based Mapping of Alternative Isoforms in the Human Proteome
Source: Cell Rep. Author manuscript; Available in PMC 2020 Jan 15. (PMC6961840; doi:10.1016/j.celrep.2019.11.026)
Supplement: 2 [file NIHMS1546469-supplement-2.zip › DF1/PXD006675/PulmonaryArtery/PulmonaryArtery_18_YWHAZ_GDEAEAGEGGERFGVSYPGIVDQSQQAYQEAFEISK.pdf]

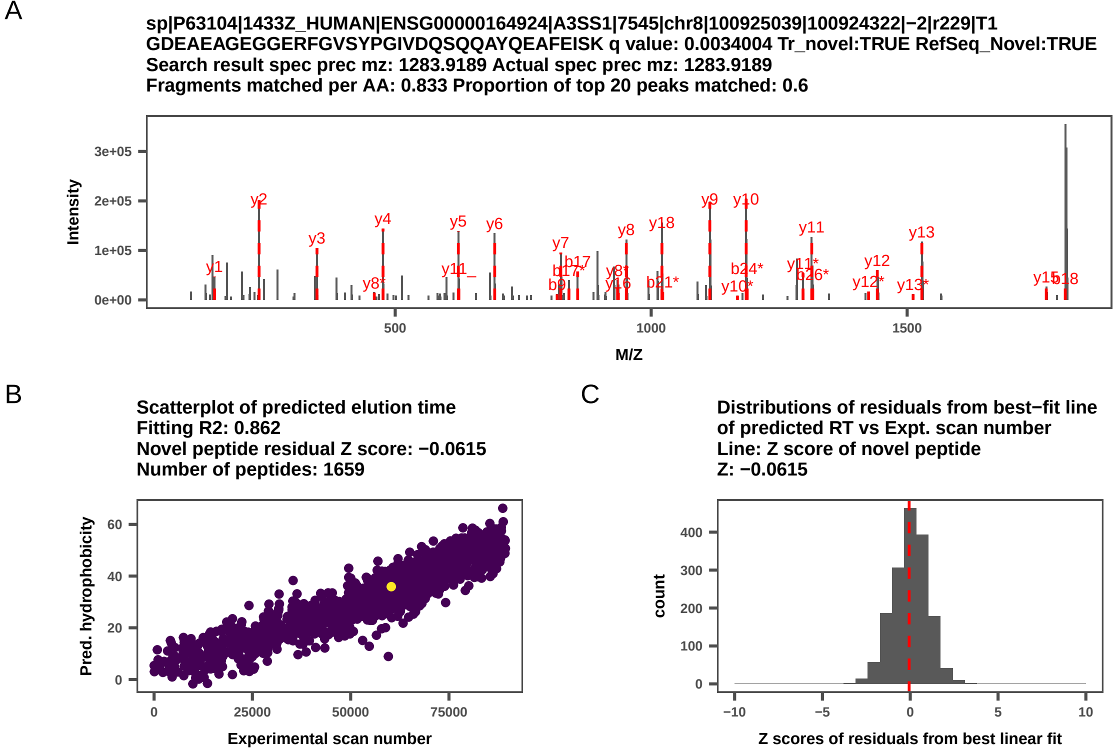
A
sp|P63104|1433Z_HUMAN|ENSG00000164924|A3SS1|7545|chr8|100925039|100924322|−2|r229|T1
GDEAEAGEGGERFGVSYPGIVDQSQQAYQEAFEISK q value: 0.0034004 Tr_novel:TRUE RefSeq_Novel:TRUE
Search result spec prec mz: 1283.9189 Actual spec prec mz: 1283.9189
Fragments matched per AA: 0.833 Proportion of top 20 peaks matched: 0.6
Intensity
3e+05
2e+05
1e+05
0e+00
y1
y2
y3
y4
y8*
y5
y11_
y6
y7
b17
b9
y8
y18
y9
y10
b21*
y10*
b24*
y11
y11*
b26*
y12
y12*
y13
y13*
500
1000
1500
M/Z
B
Scatterplot of predicted elution time
Fitting R2: 0.862
Novel peptide residual Z score: −0.0615
Number of peptides: 1659
Pred. hydrophobicity
60
40
20
0
0
25000
50000
75000
Experimental scan number
C
Distributions of residuals from best−fit line
of predicted RT vs Expt. scan number
Line: Z score of novel peptide
Z: −0.0615
count
400
300
200
100
0
−10
−5
0
5
10
Z scores of residuals from best linear fit
